# From bedside to bench: towards clinical predictive AI research that achieves real-world impact

**DOI:** 10.1136/bmjdh-2026-000044

**Published:** 2026-06-09

**Authors:** Shuqing Si, Gary S Collins, Joseph E Alderman, Ben Van Calster, Laure Wynants, Matthew R Broome, Paulina Daw, Alastair K Denniston, Joie Ensor, Benjamin I Perry

**Affiliations:** 1 Institute for Mental Health, School of Psychology, University of Birmingham, Birmingham, UK; 2 Department of Applied Health Sciences, University of Birmingham, Birmingham, UK; 3 Department of Inflammation and Ageing, University of Birmingham, Birmingham, UK; 4 Department of Development and Regeneration, KU Leuven, Leuven, Belgium; 5 Care and Public Health Research Institute, Maastricht University, Maastricht, The Netherlands; 6 Birmingham and Solihull Mental Health NHS Foundation Trust, Birmingham, UK; 7 National Institute for Health Research (NIHR) Birmingham Biomedical Research Centre, Birmingham, UK

**Keywords:** Decision Support Systems, Clinical, Patient Care, Preventive Medicine

## Abstract

Clinical predictive artificial intelligence (AI) tools, including equations or models developed using statistical, machine learning or AI methods, have proliferated, yet relatively few effectively translate into routine care. A major contributor to this bench-to-bedside gap is the lack of explicit translational planning at the outset. In this narrative review, we synthesise emerging principles for designing, developing and evaluating predictive AI tools with real-world impact in mind. We propose a pre-modelling discipline that defines the clinical question, intended users and position in the care pathway, highlights the importance of early and sustained stakeholder engagement and explicitly links programme theory to model outputs to inform decisions and change outcomes. It covers regulatory pathways and routes to self-sustainability, ethical and equity considerations and software implementation requirements that shape whether tools can be deployed and sustained. Finally, we summarise core methodological issues in model development, validation and monitoring that are particularly relevant to translation. Taken together, this bedside-to-bench approach reconceptualises clinical predictive AI tools as complex interventions, beginning with a clear expectation of their real-world deployment and benefit and planning backwards to inform model development. This shift in thinking is required to increase the chance that clinical predictive AI tools deliver real-world benefit rather than remaining confined to print.

**WHAT IS ALREADY KNOWN ON THIS TOPIC**

- Predictive artificial intelligence (AI) tools are being developed at scale, but only a small fraction are implemented or sustained in routine care, due to methodological shortcomings in model development and reporting, and a lack of translational planning.

**WHAT THIS STUDY ADDS**

- Proposes a bedside-to-bench approach in which predictive AI tools are conceived at the outset as complex interventions embedded within specific care pathways, rather than as stand-alone models.
- Introduces a pre-modelling discipline and translational roadmap, linking clinical questions and programme theory to implementation, stakeholder, regulatory, ethical and impact considerations.

**HOW THIS STUDY MIGHT AFFECT RESEARCH, PRACTICE OR POLICY**

- Encourages researchers to treat translational planning (including programme theory, implementation strategy and sustainability) as core components of predictive AI projects to support real-world applications beyond publication.
- Supports healthcare professionals and health system leaders in critically appraising predictive AI tools on statistical performance and on feasibility, governance, equity and real-world impact.

## Introduction

Clinical predictive artificial intelligence (AI) tools are models developed using statistical, machine-learning or AI-based methods to support diagnosis or predict health outcomes. Such tools range from simple integer point scores (eg, CHA₂DS₂-VASc^1^ to guide anticoagulation) to country-specific regression models (eg, the fracture risk assessment tool (FRAX^2^) to inform osteoporotic fracture prevention). With rising demand and resource constraints across health systems, strategies to reduce chronic disease burden are becoming increasingly essential.

Clinical predictive AI research offers a potential bridge between expanding availability of health data and tools that enable earlier, more targeted intervention. Research activity in the field has expanded rapidly, with an estimated 10 000 new models published annually.^3^ Ensuring this substantial research activity translates into patient and population benefit is critical.

Insufficient methodological rigour is a well-recognised translational barrier in clinical predictive AI research. During the COVID-19 pandemic, for example, prognostic models proliferated, yet almost 90% were judged at high risk of bias.^4^ Similar conclusions emerge from systematic reviews of predictive AI across biomedicine.^5–7^ Common problems include inappropriate methods, incomplete reporting of performance measures and poor adherence to reporting guidelines. In response, efforts have focused on improving methodological practices,^8^ strengthening reporting^9^ and enhancing critical appraisal.^10^ However, as methodological standards improve,^9^ other barriers along the translational pathway remain inadequately explored by clinical predictive AI researchers.

**Figure 1:**
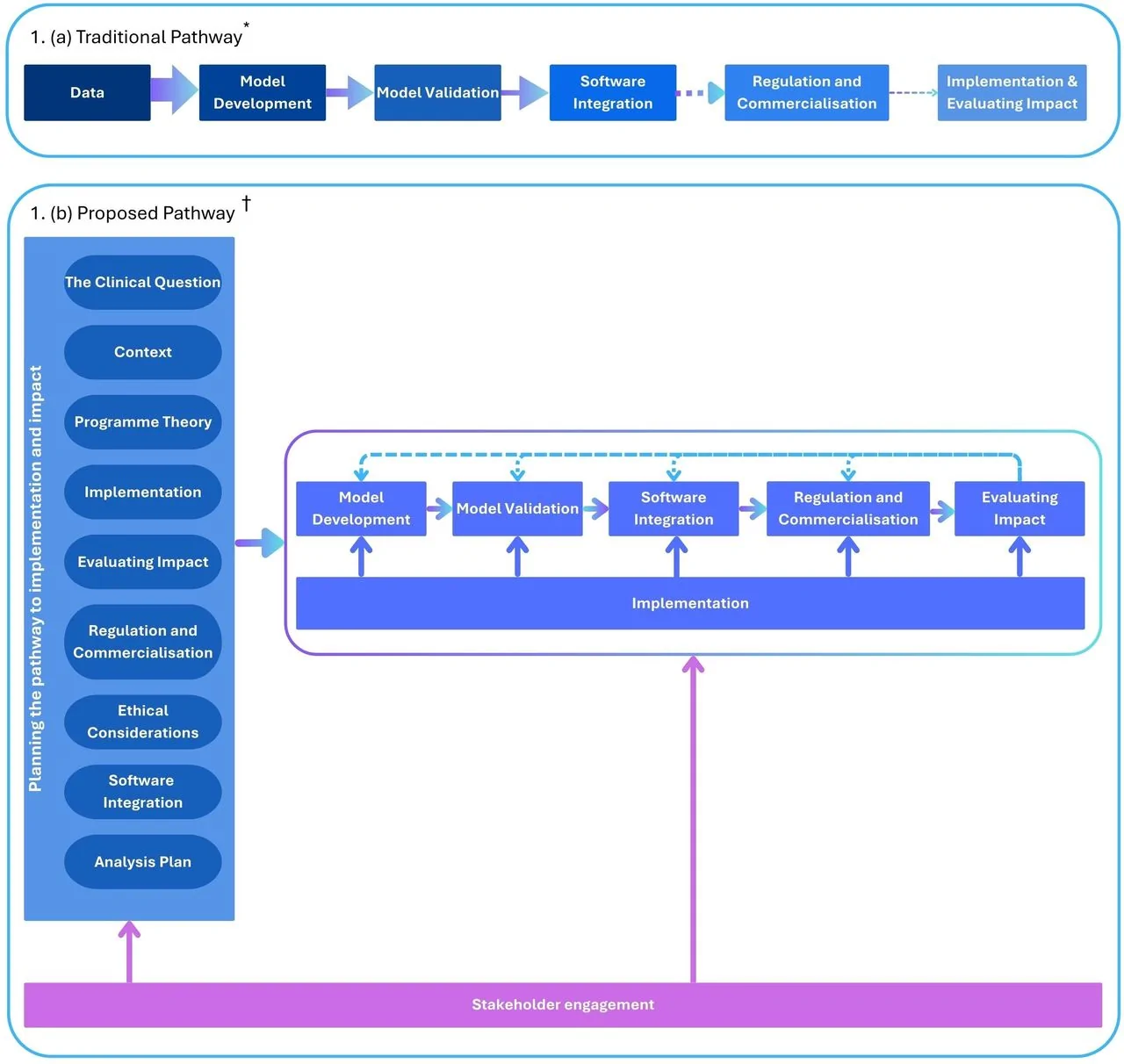
Commonly used (a) and proposed (b) workflows for developing a clinical predictive artificial intelligence tool. Further details on each component in the pathway are provided in the relevant sections of the manuscript. *The weight of gradient arrows represents the concentration of research activities at each stage. †Horizontal arrows indicate progression between translational stages and represent potential Stop/Go decision points.

The vast majority of clinical predictive AI research is concentrated at the start of the translational pathway^11 12^ and rarely progresses through a pipeline that is inherently transdisciplinary (figure 1a). Even thoughtful, well-designed studies risk becoming ‘*lost in translation*’ when downstream requirements are neglected.^13^ Successful translation demands a coordinated programme spanning clinical practice, biostatistics, qualitative research, stakeholder involvement, software/usability engineering, implementation and regulatory sciences, intellectual property management and commercialisation. A lack of infrastructure to align these components within academic settings is beginning to receive attention for how it may inhibit translational predictive AI research.^14^ While tools developed in industry settings may progress more easily as they may be better placed to support regulation and commercialisation, concerns can sometimes remain over transparency and trustworthiness.^15^

We argue that clinical predictive AI tools must not be treated as isolated statistical models, but as one component of a complex intervention that involves a wide range of interacting elements, including, for example, healthcare context, governance structures and the ways in which the tool shapes the behaviour and decision-making of patients and healthcare providers. Due to its complexity, planning a clinical predictive AI project should start with the end goal in mind—a shift to thinking from ‘bedside-to-bench’ rather than vice versa. This starts with (i) an unambiguous statement of the problem being targeted; (ii) establishing a position for the tool in existing care pathways; (iii) clear definitions of its intended purpose, intended users and intended use environment; (iv) the specific actions that should be taken in response to the tool, including any relevant timeframes and (v) plans for governance and sustainability that would enable clinical use. Developers can then work back to context-appropriate data sources and analyses (figure 1b), and recognise that these stages might be refined as preliminary evidence emerges. This pre-modelling discipline makes programme theory explicit, clarifies how benefit will be achieved and measured and provides clear direction on appropriate upstream statistical and methodological choices. This review sets out the translational roadmap.

## Stakeholder engagement

Stakeholders, those who can influence design, adoption or impact, are critical to delivering impactful clinical predictive AI research. Engaging a broad range of stakeholders across the translational pipeline ensures that tools are designed around real-world problems and user needs, promoting relevance, acceptability, feasibility and ultimately impact in real-world settings.^16^

While stakeholder engagement is becoming more common in biomedical research, it remains inadequately considered in most clinical predictive AI research.^17 18^ Neglecting stakeholder engagement until the point of implementation risks making upstream decisions that will inhibit successful adoption and impact. For instance, in post-market qualitative evaluations of CanRisk, a tool that predicts breast and ovarian cancer risk, general practitioners reported that completing the online tool exceeded typical appointment duration,^19 20^ potentially limiting CanRisk’s usefulness for a key intended user base. Earlier and more intensive stakeholder engagement might have mitigated these design barriers.

Encouragingly, awareness is starting to grow. The recently updated Transparent Reporting of a multivariable prediction model for Individual Prognosis Or Diagnosis+Artificial Intelligence (TRIPOD+AI) reporting guidelines for clinical prediction models underscore the importance of patient and public involvement and engagement.^9^ However, stakeholders are broader than patients and the public alone, and also include healthcare professionals, healthcare organisations, IT/governance teams, regulators/policymakers and industry. Beyond identifying a broader range of stakeholders, the methods used for engagement must also be carefully considered. For example, to reduce power imbalances between stakeholder groups, meetings or focus groups may be conducted separately for different stakeholder groups. Where mixed group discussions are preferred to improve shared understanding, facilitators must seek to promote equality between participants, for instance, through the use of ground rules and/or actively encouraging all attendees to contribute equally. Researchers should also consider strategies to improve accessibility so that engagement activities comprise representative samples of the target stakeholder population. In addition, questions and engagement approaches may need to be tailored to different stakeholder groups to maximise the relevance of feedback. Finally, research teams should be transparent about any potential project limitations so that stakeholder expectations align with what is feasible.

Levels of stakeholder involvement in predictive AI projects are likely to vary by project and phase. Different levels of engagement range from passively receiving information to being integrated into a research team to co-produce research outcomes.^16 21 22^ Applying this framework to the development of predictive AI tools, levels of engagement could be defined as follows: (i) *consultation*, where stakeholders provide feedback or advice; (ii) *collaboration*, where stakeholders work in ongoing partnership with input systematically incorporated into decisions and (iii) *co-production*, where stakeholders act as equal partners with joint ownership over design, implementation and evaluation. Used in isolation, consultation may not be sufficient and risks being perceived as tokenistic.^23^ Genuine co-production is rare and resource-intensive,^24 25^ but is associated with improved research practices and outputs.^26^

**Figure 2:**
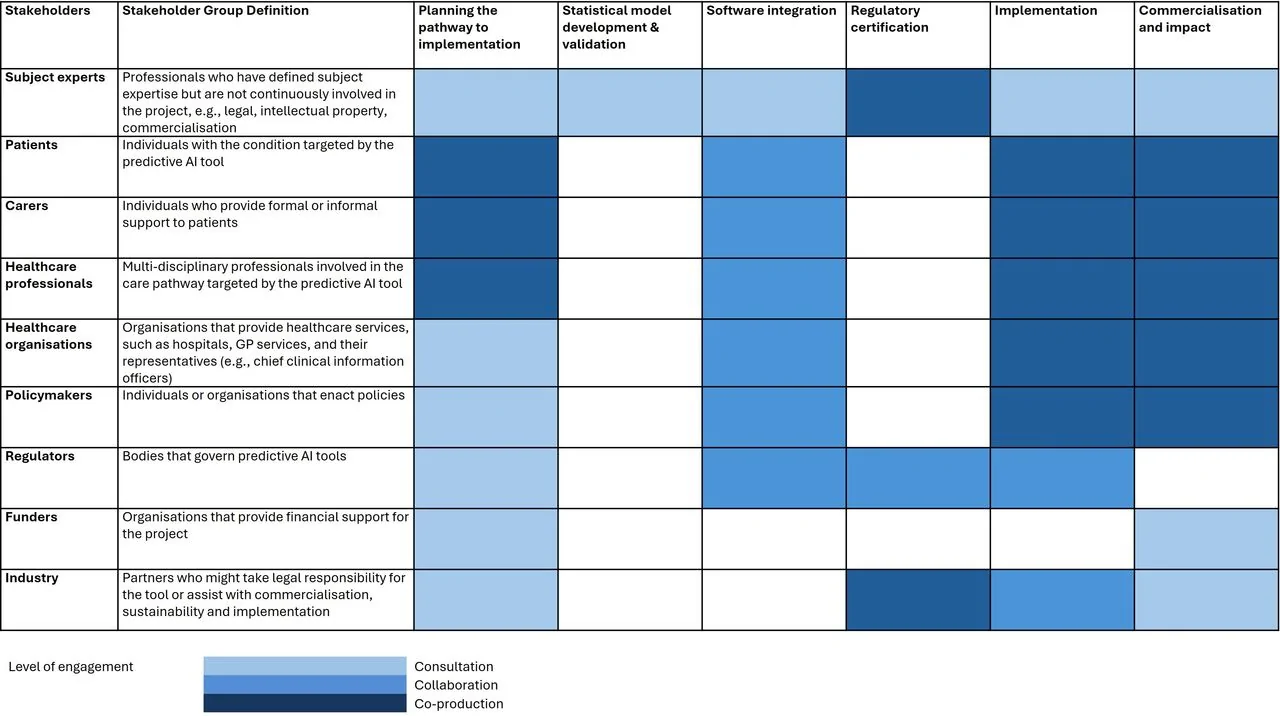
Translational clinical predictive AI template for stakeholder engagement as applied to PsyMetRiC. AI, artificial intelligence; GP, general practitioner.

PsyMetRiC, a predictive AI tool for cardiometabolic risk in people with psychotic disorders, is designed to support clinician-patient communication and shared decision-making, so close collaboration with patients, carers and clinicians was essential.^27^ For a clinician-operated automated hospital sepsis alert system (table 1, example 2), early involvement of clinicians, informatics teams and governance leads may take precedence. Whatever the configuration, stakeholder groups should be involved early in planning and design (see the ‘Planning the pathway to implementation and impact’ section). Figure 2 illustrates a stakeholder engagement plan for PsyMetRiC.

## Planning the pathway to implementation and impact

Inspired by complex intervention frameworks,^28^ we contend that predictive AI tools are multicomponent systems. The model is inseparable from its software/user interface, regulatory and technical documentation, monitoring and maintenance plans and any decisions triggered by its outputs. These systems are inseparable from their context (population, users, workflow, governance and sustainability) (see the ‘Regulation and commercialisation’ section). A clear programme theory should underpin research activities from the outset and be revised as the project proceeds. Programme theory should specify mechanisms, context and how adoption and impact will be assessed.

### The clinical question

A precise, clinically meaningful question is the strategic foundation of any impactful clinical predictive AI tool. The PICO (Patient/population, Intervention, Comparison, Outcome) and FINER (Feasible, Interesting, Novel, Ethical, and Relevant) frameworks^29^ have highlighted the importance of targeted population, outcome definition, feasibility, ethical, impact considerations predictive AI research requires additional consideration of label construction, temporal horizons, actionability of outputs and risk of bias or misalignment with clinical decision-making. Rather than starting from convenience with available data or favoured modelling technique, planning must specify the unmet clinical need and the point at which prediction could plausibly inform decision-making to improve outcomes. This means specifying who decides what, when and how predictions improve decisions versus current care. Therefore, value depends not merely on statistical performance, but on whether outputs trigger meaningful actions in response. For example, in UK primary care, QRISK3, a population-based tool estimating 10-year cardiovascular risk. The underlying research question is whether the risk of cardiovascular disease within the next 10 years can be predicted among adults in the general population who are not currently receiving statin treatment. And the prediction outcome is directly linked to evidence-based preventive pathways.^30^ By contrast, while an accurate model predicting risk of developing psychosis may have limited preventive value given the current shortage of effective preventive interventions, such a model may still have value in communication or care planning.

After establishing a meaningful clinical question, a systematic review of existing tools is an appropriate next step. Mapping existing tools can identify gaps in use, feasibility and performance. On this evidence, a decision can be made on whether the best route is adoption or adaptation of an existing tool, or de novo development.^13^

### Context

Contexts in clinical predictive AI research are multilevel and dynamic, spanning (i) deployment; (ii) users and use setting and (iii) the regulatory and commercial environment. Clinical predictive AI tools can change referral thresholds, testing patterns and the behaviour of clinicians or patients, with knock-on effects on workload, costs and equity. Anticipating feedback effects and monitoring for unintended consequences should be built into the programme theory and revisited through the project lifecycle.

#### Deployment context

Defining the target population must include considerations for the potential heterogeneities that exist within it. For instance, clinical predictive AI tools developed for the broad general population often perform less well in specific subgroups,^31 32^ potentially propagating health inequities. Where this risk is identified, subgroup-specific models could be explored as a potential approach. However, this approach must be considered carefully on a case-by-case basis. For instance, subgroup sample sizes are likely to be smaller, which may increase model uncertainty, and data representativeness may not be even across subgroups. Both of these factors may contribute to worsened health inequalities if such models are implemented. Predicted outcomes should also be clinically meaningful and actionable. The timing and decision that the tool is intended to support must be clearly articulated—clinical predictive AI is only likely to be impactful when its output is tied to a well-defined step in the workflow.^33^ More considerations in deployment context are discussed in later relevant sections.

#### Users and use setting

The tool should be designed with the intended user in mind. In primary care, tools should not be burdensome or time-consuming to complete and should rely on information that is readily available or easily collected. In specialist settings, richer inputs will be more feasible. Healthcare professional-facing tools might assume better familiarity with clinical variables and any decision thresholds. Conversely, the ‘Know Your Risk’ diabetes calculator, based on the Leicester Risk Assessment score, is public-facing, avoids laboratory tests and uses accessible language.^34^

Researchers must be clear from the outset whether the tool is intended for clinical use. In psychiatry, for instance, neuroimaging studies have identified potentially valuable predictive biomarkers,^35^ but imaging data are neither routinely nor easily collected in practice. Such tools may have greater value in research settings, for example, to guide trial recruitment and stratification in the evaluation of new interventions. In these cases, the primary users of predictive AI tools developed using research-grade predictors will be researchers rather than healthcare professionals.

Finally, scope, whether the tool is intended to be used locally, regionally, nationally or internationally, should shape design, implementation and evaluation. A tool developed within a single locality may require recalibration or updating when applied elsewhere, alongside flexible implementation strategies to accommodate heterogeneous care pathways.^36 37^

#### Governance

Clinical predictive AI tools are deployed within healthcare systems governed by regulation, procurement processes and budget constraints. These external conditions shape what can realistically be built and sustained, because most tools carry ongoing obligations after release. These include version control, surveillance/incident handling, cybersecurity and change control. Regulatory requirements are closely linked to the tool’s intended use, which in turn determines its risk classification and the scale of evidence, quality management and post-market activities expected. Therefore, intended use should be defined pragmatically to be clinically meaningful while also compatible with the available resources required to maintain the tool (see ‘Regulation and commercialisation’ section). Considering these constraints early is therefore part and parcel of designing for impact.

### Programme theory

In keeping with complex-intervention thinking,^28^ programme theory explains how a clinical predictive AI tool can achieve impact, and how impact interacts with context. It provides a conceptual map linking the components of the tool, mechanisms of action, contextual interactions and expected outcomes.^28^ Programme theory should be considered early in the planning stage, and revisited and refined as evidence accumulates.^38^

In clinical predictive AI research, programme theory might propose that if clinicians and patients are provided with risk estimates at appropriate decision points, then clinical management or health behaviours may be informed and modified in ways that alter health outcomes. It also prompts explicit consideration of contextual factors—workflow integration, usability, organisational support and potential negative consequences.^39 40^ As such, programme theory can provide the theoretical foundation for bridging statistical performance with real-world impact.

**Figure 3:**
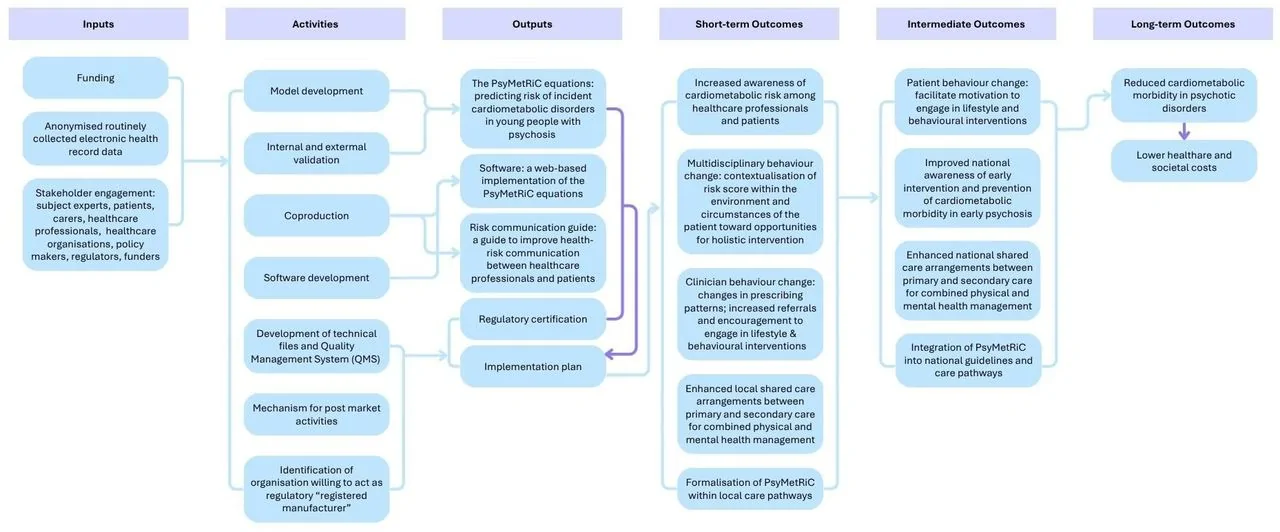
Example of a logic model for PsyMetRiC—a clinical predictive artificial intelligence tool for cardiometabolic outcomes in young people with psychosis.

Logic models are a common way to document programme theory. They are diagrams that provide visualisation of the relationships between key components of an intervention, including inputs, activities, the short-term and long-term outcomes, helping researchers to consider how different components of the intervention interact to provide intended effects.^40 41^ We provide an example of a logic model for PsyMetRiC in figure 3.

### Implementation

An early implementation plan forces researchers to design for deployment, not just publication. It clarifies whether a tool could be used, by whom, where in the pathway and with what support, making readiness a precondition for impact evaluation and helping to avoid investment in infeasible models.^42^

Although most implementation frameworks were not originally developed specifically for clinical predictive AI tools, several offer complementary guidance and could be applied together. PRISM (the Practical, Robust Implementation and Sustainability Model)^43^ and RE-AIM (Reach, Effectiveness, Adoption, Implementation, and Maintenance)^44^ emphasise reach, effectiveness, adoption, implementation and maintenance, while keeping attention on sustainability. The NASSS (Nonadoption, Abandonment, and Challenges to the Scale-Up, Spread, and Sustainability) framework^45^ embraces complexity across technologies, organisations and systems. The SALIENT framework^46^ proposes a staged pathway from development and validation to deployment and monitoring. The FAIR-AI (Framework for the Appropriate Implementation and Review of AI)^47^ adds governance for assessment, approval and oversight. Together, these frameworks suggest a hybrid, staged plan linking programme theory to concrete choices about integration, measurement governance and sustainability.

Alongside existing implementation frameworks, several additional practical considerations are required for clinical predictive AI tools. Computational requirements should be assessed early, including whether underlying algorithms could be simplified (eg, point scores/nomograms) for offline or low-resource settings. Cost and resource are also central to implementation planning. Staff training may promote correct and consistent use, facilitating fidelity, but increases costs and may prolong scale-up.

Finally, implementation may occur in a bottom-up or top-down manner. Fashioning and iterating a local implementation strategy within a single setting or region before scaling may enable deep consideration of implementation facilitators and barriers but is time-consuming. Conversely, aiming for an immediate implementation strategy (eg, through inclusion in national clinical guidelines) may enable scaling at pace, but may limit potential to explore implementation factors in depth.

### Evaluating impact

Achieving an arbitrary level of statistical performance does not guarantee real-world impact of a clinical predictive AI tool.^48 49^ Statistical measures such as net benefit from decision curve analysis aim to bridge this gap,^50 51^ but are blind to context and programme theory, and therefore to the feasibility of impact. Real-world evaluation should explore what types of impact the tool may generate (positive and negative), through which mechanisms, under what conditions and with what broader consequences, mirroring evaluation of complex interventions.^42^ Impact studies in predictive AI research are concerningly scarce, including for tools that are currently used routinely in clinical practice.^52–54^

The choice of outcomes is an important consideration in impact evaluation and should be determined collaboratively with stakeholders. Relevant domains may include:

1. Clinical outcomes (eg, diagnosis, referrals, complications).
2. Behavioural outcomes (eg, clinician or patient behaviour).
3. System-level outcomes (eg, resources, workflow, policy).
4. Clinical outcomes (eg, diagnosis, referrals, complications).

The potential for unintended and negative outcomes should be explicitly considered, including clinician over-reliance, patient anxiety or stigma or inequity. Impact evaluation may also reveal shortcomings in upstream components of the translational pathway, informing iterative refinements in research processes (figure 1b). Randomised controlled trials may be infeasible or inadequate to fully capture the multifaceted impact of predictive AI. Mixed-methods, before-after and time-series designs can provide valuable insight.^55^

## Regulation and commercialisation

Global healthcare regulators typically define clinical predictive AI tools as medical devices, necessitating a range of pre-market and post-market considerations before and after clinical deployment.^56^ Meeting and maintaining regulatory certification demand ongoing commitment across the tool’s lifetime. Without a realistic plan for how this will be resourced, even accurate and equitable tools are unlikely to be adopted or sustained in clinical practice.

The global regulatory landscape is heterogeneous, and the same tool may be classified differently across jurisdictions. For instance, under the EU Regulation (EU) 2017/745 (EU MDR), most clinical predictive AI tools that provide information used to make diagnostic or therapeutic decisions fall into at least class IIa.^57^ In Great Britain, the Medicines and Healthcare products Regulatory Agency currently operates under the UK Medical Devices Regulations 2002 (as amended), where many comparable tools remain class I, although reforms are in progress.^58–60^ In the USA, clinical predictive AI tools are regulated by the Food and Drug Administration, which excludes certain clinical predictive AI tools from regulatory frameworks if they meet specific non-device clinical decision support criteria.^61^

The International Medical Device Regulators Forum has proposed a reference risk categorisation matrix that considers both the degree of influence of the software on clinical decision-making and the severity of the condition.^62^ This framework up-classifies some clinical predictive AI tools compared with international jurisdictions, while down-classifying others. We summarise potential cross-jurisdictional classifications in table 1.

**Table 1:** Worked examples of regulation of three predictive AI tools in different global regulatory frameworks

Jurisdiction	Possible classification	Relative resource implications	Rationale for possible classification
Example 1: a regression-based cardiovascular prediction model providing estimates of 10-year risk of a heart attack or stroke, implemented as a web-based tool used in primary care			
Great Britain (UK MDR)	Class I	Low	Stand-alone software with a medical purpose; most classified as class 1
European Union (EU MDR)	Class IIa	High	Rule 11 for software (provides information with which to take therapeutic decisions) classifies as minimum class IIa.
USA (FDA)	Exempt	Lowest	If all four statutory criteria are met, it may fall outside of the regulatory framework.
IMDRF (International Reference)	Category I	Low	Based on levels of healthcare situation/condition and information provided by the model, it matches category I in risk grid. Note this could be category II if risk thresholds dictate specific interventions (direct clinical management×serious condition).
Example 2: a gradient-boosted machine learning model providing early warning for sepsis in an acute hospital setting, implemented as an automatic alert and treatment recommendation system for clinicians			
Great Britain (UK MDR)	Class IIa–b	High	Supports diagnostic/management actions where harm could be serious.
European Union (EU MDR)	Class IIb	High	Rule 11: IIb applies when erroneous output could cause serious deterioration.
USA (FDA)	Class II	High	Typically class II for opaque models that are not eligible for the non-device CDS route.
IMDRF (International Reference)	Category III	Highest	Drives clinical management×critical context matches category III in risk grid.
Example 3: a deep-learning model that triages CT images for suspected ICH, implemented automatically within a health record software, flags likely ICH and prioritises triage speed by health professional			
Great Britain (UK MDR)	Class IIb	High	Stand‑alone active diagnostic/triage software; higher class expected given potential for harm.
European Union (EU MDR)	Class IIa–b	High	Rule 11 for acute, potentially irreversible harm; in some circumstances, class III could apply.
USA (FDA)	Class II	High	Image analysis (not eligible for non-device CDS route).
IMDRF (International Reference)	Category III	Highest	Drives clinical management (triage)×critical condition matches category III in risk grid.

These are possible classifications for illustration only. Actual classification depends on the precise intended purpose, claims, user population and foreseeable harms. Consulting with regulatory experts is critical for correct classification.

AI, artificial intelligence; CDS, clinical decision support; FDA, Food and Drug Administration; ICH, intracranial haemorrhage; IMDRF, International Medical Device Regulators Forum; MDR, Medical Devices Regulations.

Regardless of jurisdiction, initial resources will be required to bring a clinical predictive AI tool into practice, and ongoing resources will be necessary to sustain it. Since most clinical predictive AI research arises from academia, early engagement with institutional commercialisation, technology transfer and intellectual property teams is essential to establish self-sustaining pathways. These may include reimbursement, licensing or service contracts.

## Ethical considerations

Predictive AI research raises ethical questions that bear directly on safe, trustworthy use. While there is currently no ethical guidance specific to clinical predictive AI research, WHO has published guidance for AI for health in general.^63 64^ These and other frameworks are useful reference points, although adherence is voluntary and compliance is not presently independently assessed. Here, we highlight selected examples with relevance to clinical predictive AI research.

### Transparency, explainability and intelligibility

Transparency is essential when a tool’s logic, inputs and limitations are opaque; otherwise, healthcare professionals may struggle to explain the basis of their decisions to patients adequately.^65^ The TRIPOD+AI guideline^9^ provides a reporting framework to support transparent development and reporting of predictive AI tools.

### Fostering responsibility and accountability

If a recommendation derived from a clinical predictive AI tool contributes to harm, responsibility may be disputed between healthcare professionals, developers and deploying organisations. Mitigations include documentation, auditability and clear accountability structures.

### Protecting autonomy

Agency is an important consideration in predictive AI research, beyond its potential association with regulatory risk classification (see ‘Regulation and commercialisation’ section). Predictive AI tools that dictate clinical actions may reduce agency and shared decision-making capacity. When risk scores are auto-generated within electronic systems and presented without discussion, patients may have little influence over whether or how those predictions shape their care. Because risk information can heighten anxiety, early engagement with patients should elicit preferences regarding if, when and how such information should be communicated, as well as what forms of support or resources should accompany it. Risk communication is inherently challenging. Both patients and professionals may misinterpret risk estimates,^66^ although accessible information can improve understanding.^67^ Communication strategies should therefore be designed to maximise understanding and promote meaningful dialogue about health risks.^27^

### Equity and the promotion of human well-being, safety and the public interest

Clinical predictive AI tools reflect the data on which they are built; when certain populations are under-represented or have unequal access to care, their records may be sparse or missing,^68 69^ and may reflect underdiagnosis or undertreatment. This risks embedding existing healthcare inequalities into tools and perpetuating them.^70^ Evaluating predictive performance across relevant subgroups is an important step, with results being transparently reported. However, any steps taken to minimise inequity must be rationalised and considered carefully. Ascertainment bias due to unequal healthcare access may be difficult to quantify, and there are clear examples where modelling strategies intended to reduce inequalities have instead exacerbated them.^71^ Stakeholder-informed mitigations outside the data, such as subgroup-specific thresholds or monitoring strategies to prioritise equitable access, may sometimes be more appropriate.

## Software integration

Most predictive AI research generates equations or models that require software implementation before they can be used in practice. Two routes are common: integration within existing healthcare platforms or development of stand-alone software. Each has strengths and limitations.

Integration can automate scoring and embed outputs in routine systems, reducing workload. However, integrated tools that are permanently active may increase reliance, constrain independent clinical judgement and contribute to alert fatigue from multiple, repeated or false positive prediction alerts. Systems should therefore be designed carefully to ensure that multiple predictive AI tools are implemented in a way that avoids overwhelming the user and ensures alerts are trusted and actionable.^72^ Integration may also restrict design flexibility and may require the host software to undergo regulatory certification itself .

Stand-alone software or web-based tools provide greater design flexibility and promote co-production. This enables interfaces tailored to the model, user needs, target population and clinical workflows, potentially enhancing both understanding and communication. However, adoption may be more challenging and integration into workflows less straightforward.

In all cases, regulators scrutinise the software implementation as the medical device, rather than the underlying model in isolation. Software should be developed under an appropriate Quality Management System, incorporating clear design requirements (intended purpose, core functions and user interface), a comprehensive risk analysis and mitigation plan, and both formative and summative usability testing to ensure the software meets user needs and can be safely integrated into clinical workflows. A post-market surveillance plan is required to monitor the software over time, document incidents with clear processes to address them and guide ongoing maintenance and updates.

## Model validation

External validation is critical to evaluate generalisability and is essential for any tool intended for use beyond the development setting.^48 49^ Unlike internal validation, which assesses reproducibility within the development population, external validation evaluates performance in a different but related population. Models that perform well internally will often show poorer calibration and discrimination performance when applied elsewhere.^48^ External validation studies are important to understand the extent to which predictor-outcome relationships remain stable across case mixes, healthcare systems, measurement methods and demographics. Where differences are identified, researchers might consider approaches such as updating, recalibration or the development of population-specific versions. External validation studies, despite their importance, remain relatively rare across biomedical predictive AI research.^73 74^

The process of external validation confronts predictive AI researchers with a dilemma: balancing generalisability and transportability with the need for tools that are accurate and equitable for diverse subpopulations and settings. Achieving both may be extremely challenging. No tool can ever be considered ‘*fully validated*’. External validation must be repeated as populations and care practices evolve, although conducting validation *after* a tool is deployed brings its own challenges.^75^ These considerations in evaluation should, in turn, inform model development, underscoring the importance of design with downstream requirements in mind.

## Model development

Model development is often treated as the starting point of clinical predictive AI research, but within a translational framework it should be guided by downstream considerations. Decisions about outcomes, predictors and modelling steps should be grounded in maximising the chances of successful translation. Data sources should be chosen not merely on convenience, but for relevance and similarity to the intended use setting. Where appropriate data sources for development and downstream validation are not available, researchers should think carefully about whether the project should be commenced. Detailed guidance on statistical methodology for predictive AI model development can be found elsewhere.^8 48 76^

Model development is also shaped by the quantity, structure and representativeness of available data. Datasets should be sufficiently large relative to the number of proposed predictors to minimise overfitting and produce stable estimates.^8 77^ Principled sample size methods^8 78^ and software can guide planning. Pragmatism may be required to align the models with the available data. For instance, PsyMetRiC aimed to include a measure of insulin resistance, a key abnormality detectable in early psychosis as a predictor, but as it is not routinely measured, triglycerides and high-density lipoprotein cholesterol were included instead as a proxy.^79^ Missing data are a certainty in health datasets and may also shape modelling choices. A range of approaches exist to mitigate the impact of missingness. Developers should consider whether missing data will be permitted at deployment and ensure that corresponding strategies are incorporated during model development.^80^

Internal validation using bootstrapping or cross-validation is also an important part of the development process,^48^ for instance, to guide coefficient shrinkage and assess stability.^37 48^

## Conclusion

Clinical predictive AI tools are widespread in biomedical research, yet few progress beyond publication to the point of adoption into clinical use. Bridging this bench-to-bedside gap requires a shift in perspective: models should not be conceived in isolation, but as complex interventions embedded within specific clinical workflows, organisational structures and regulatory environments.

Planning should start at the bedside: first, understand the health need, define the clinical question that the model seeks to address, determine the position in the care pathway, map and engage stakeholders and develop the programme theory. Then align model development, evaluation, software implementation and regulatory strategy to this blueprint. Ethical and equity considerations, alongside realistic plans for governance and sustainability, must be integrated from the outset rather than as a downstream addition.

The development of clinical prediction AI tools is transdisciplinary and requires dedicated systematic infrastructure support. However, by adopting this bedside-to-bench approach—designing for implementation and impact from the beginning—clinical predictive AI research can move beyond adding to an already saturated literature and instead deliver tools that are of sufficient quality to enable more equitable, effective and efficient care. The approach can also inform the use of Stop/Go criteria when planning more ambitious or translational predictive AI projects, helping to avoid unnecessary waste of resources and mitigating risks during project development. Stop/Go checkpoints may be embedded between each translational stage (figure 1).
